# Supplementary material for: Reconfigurable photonics with on-chip single-photon detectors
Source: Nat Commun. 2021 Mar 3;12:1408. doi: 10.1038/s41467-021-21624-3 (PMC7930283; doi:10.1038/s41467-021-21624-3)
Supplement: Supplementary file 1 — Supplementary Information [file 41467_2021_21624_MOESM1_ESM.pdf]

Supplementary information
Reconfigurable photonics with on-chip single-photon detectors

Samuel Gyger,[1,*] Julien Zichi,[1] Lucas Schweickert,[1] Ali W. Elshaari,[1] Stephan Steinhauer,[1] Saimon F. Covre da Silva,[2] Armando Rastelli,[2] Val Zwiller,[1] Klaus D. Jöns,[1] and Carlos Errando-Herranz[1,†]

[1] *Department of Applied Physics, KTH Royal Institute of Technology, Stockholm, Sweden*
[2] *Institute of Semiconductor and Solid State Physics, Johannes Kepler University Linz, Linz, Austria*

I. SAMPLE DESCRIPTION

Scanning electron micrographs (SEMs) of our device can be found in Fig. 1b and Fig. S1. The waveguides are 250 nm thick and 500 nm wide, with SiO_2 bottom cladding and air top cladding, adiabatically coupling into 400 nm wide, air–clad waveguides in the directional coupler region. The waveguides bend at a radius of 25 µm, which was designed conservatively to avoid optical radiation loss. The grating couplers are air–clad, and have a period of 900 nm and a duty cycle of 50 %, and were designed for coupling into a microscope objective following previous literature [1], and optimized in previous fabrication runs by sweeping and measuring grating transmission, which we measured to be around 10 % for a microscope objective with a NA of 0.65. Near the SNSPD, the waveguide width tapers to 550 nm. The SNSPD is fabricated in a 9 nm thin NbTiN film, and features a hairpin design with 65 nm width (the measured width of the SNSPD nanowire is 90 nm) and 40 µm total nanowire length. The MEMS actuator is a cantilever of length 9.5 µm and width (i.e. directional coupler length) 80 µm, with 1 µm diameter holes for hydrofluoric acid wet under–etching. The anchors to connect the suspended waveguides to the rest of the chip widen to 650 nm with a 1.5 µm long taper, followed by a 1.5 µm straight section before tapering down symmetrically. The 6 clamps connecting the MEMS actuator to the tapered waveguide are 300 nm wide and 1.6 µm long, and are separated with a pitch of 23.5 µm. The directional coupler was designed with symmetrical cantilevers, with only one cantilever electrically contacted to act as the MEMS actuator.

Figure S2 shows close-up SEM images of the tip of the hairpin of our two SNSPDs. While Detector A shows no apparent defects, Detector B shows a constriction at the turn of the hairpin. We observed no constrictions in other SNSPDs in closer proximity to other MEMS structures on the same chip, and thus we attribute this defect to a lithography error.

II. SAMPLE FABRICATION

The process flow of the sample fabrication is shown in Fig. S3. We expect the BHF process to etch the thin native oxide film on the surface of the NbTiN, self-limited to 1.3 nm [2]. This oxide film would grow again after exposure to atmospheric oxygen, resulting in a slightly thinner superconducting film [2]. The reduction in thickness, well below a nanometer, might result in a slight degradation of the superconducting properties of the exposed areas of our 9 nm NbTiN film [2]. However, in our fabrication process, our SNSPDs were protected by resist, and thus we observed no degradation of their properties.

Previous to our MEMS measurements, we characterized SNSPDs from a cleaved portion of the same chip in a closed-cycle cryostat at 2.5 K, which yielded saturation of internal quantum efficiency at 850 nm for 3 out of 5 tested devices (Figure S4). All of the detectors are identical in SNSPD design, including waveguide integration, while the green and orange curves are from SNSPDs with MEMS actuators similar to the device reported in the main text.

III. SNSPD DESIGN AND SIMULATIONS

We performed simulations of waveguide–coupled SNSPD with an eigenmode solver (COMSOL Multiphysics) using the NbTiN material parameters in Banerjee et al. [3]. For our fabricated waveguide cross-section, the simulated absorption is $-0.675\,\mathrm{dB\,\mu m^{-1}}$. Our SNSPDs are 20 µm long, yielding an absorption of $-13.5\,\mathrm{dB}$, or 95.5 %.

* gyger@kth.se
† carloseh@kth.se

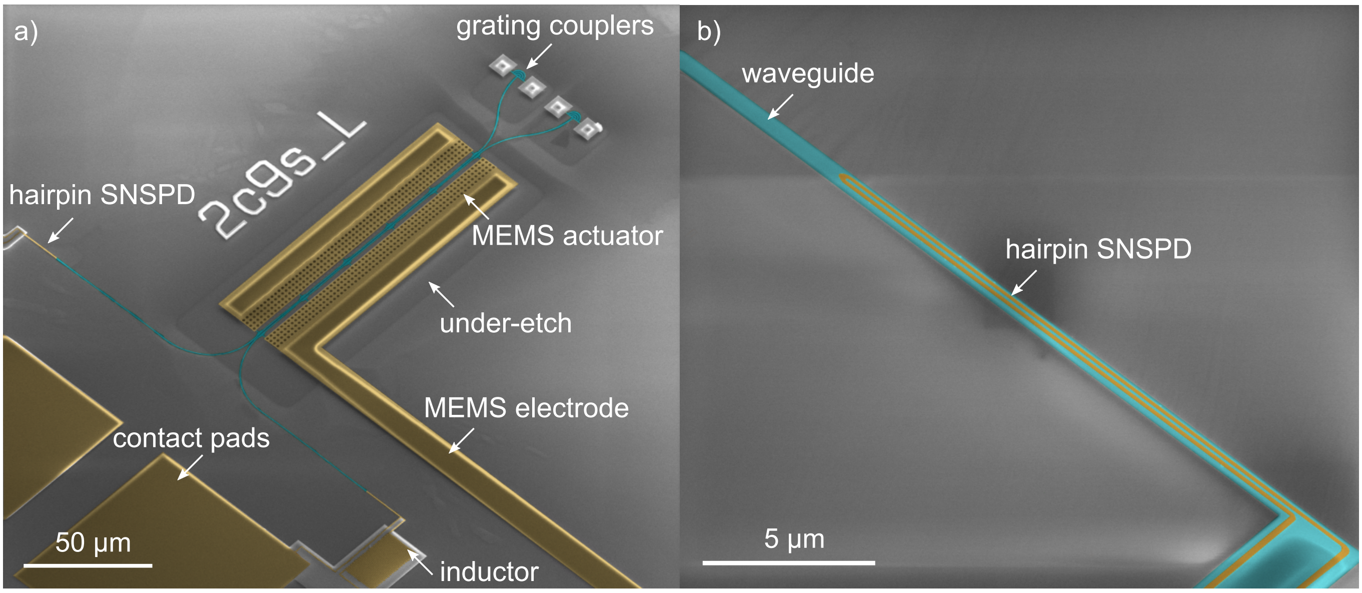

FIG. S1. SEM images of our device. a) Full view including the two input grating couplers, the suspended directional coupler and routing waveguides (blue) and MEMS actuators, electrodes, SNSPDs and contact pads (gold). b) Close–up of one of the waveguide–coupled SNSPDs.

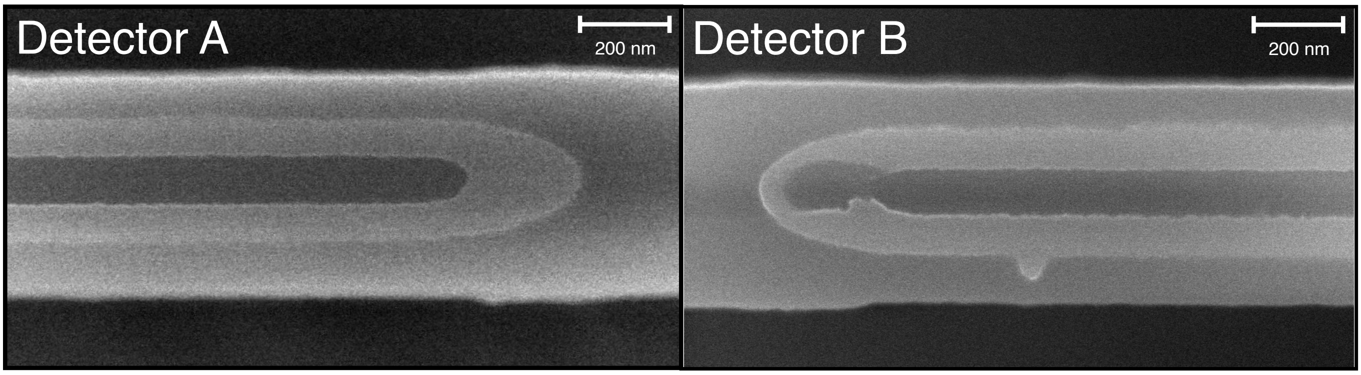

FIG. S2. SEM images of the SNSPD hairpins. It is visible that Device B has a defect in the bend leading to a low critical current, limiting the detection efficiency of the straight section.

IV. SNSPD CHARACTERIZATION

We measured the photon count rate depending on bias current of the SNSPDs through the waveguide at 795 nm. The curves were individually normalized to the saturation plateau of our devices. From the power meter measurements, we calculate a system detection efficiency from the cryostat window to the detector of -33.7 dB for detector A and -49.3 dB dB for detector B, when all the power is routed to the respective detectors.

The critical currents (current densities) in the straight section are 15.8 µA (19.5×10^9 A/m^2) for Detector A and 5.9 µA (7.3×10^9 A/m^2) for Detector B. The critical current of Detector B is limited by the defect visible in Fig. S2 with an estimated critical current density in the constriction (width of 35 nm) of 18.7×10^9 A/m^2 similar to Detector A in the straight section.

The devices show a reset time given by the Gaussian decay fit of 4.73 ± 0.03 ns for detector A, and 4.73 ± 0.01 ns for detector B, shown in Fig. S5.

We measured the jitter of our integrated SNSPDs using a 2 ps pulsed laser system at 795 nm using room temperature amplification in the commercial driver system. The photo-diode generated trigger signal and the SNSPD signal were correlated on a 40 GS/s, 4 GHz bandwidth oscilloscope (WaveRunner 640Zi, LeCroy). The extracted jitter from an exponentially modified Gaussian fit is 121 ps for detector A and 253 ps for detector B (see Fig. S6). The detectors were biased at $0.8I_c$ with 36 dark counts per second (detector A) and $0.88I_c$ with 25 dark counts per second (detector

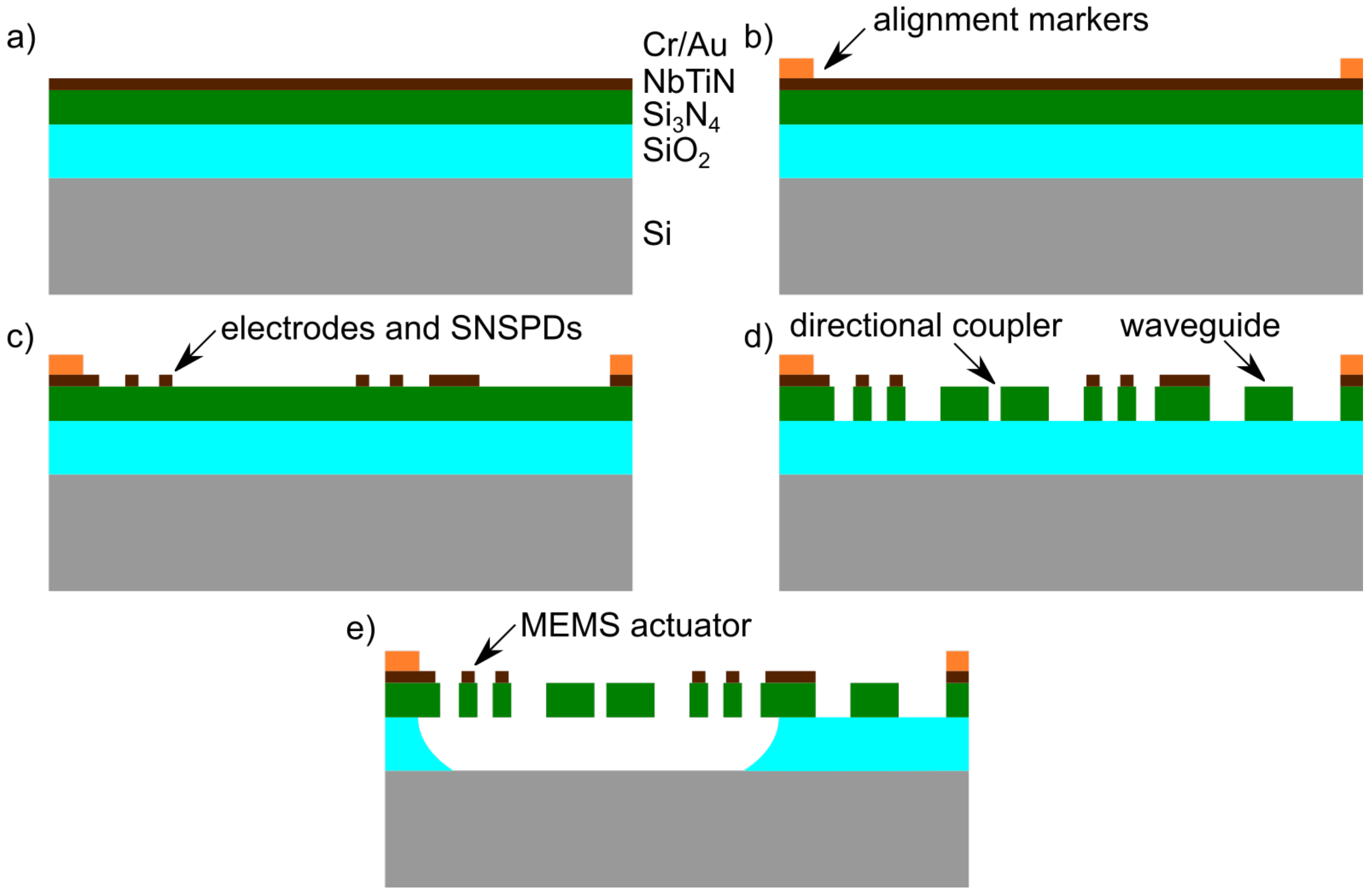

FIG. S3. Cross-section schematic of the fabrication process for the MEMS reconfigurable device with SNSPDs. a) Deposition of NbTiN on a foundry–grown $Si_3N_4/SiO_2/Si$ substrate, followed by b) Au/Cr lift–off of markers, c) NbTiN and d) Si_3N_4 patterning using electron beam and reactive ion etch, and e) BHF under–etching through resist windows patterned via photolithography followed by CPD.

B).

The absolute detector counts in Fig. 1c are 190472 counts per second (Detector A), and 45955 counts per second (Detector B) for a DCR of 100 counts per second.

For the MEMS characterization in Fig. 1d, our absolute measured counts ranged from 4 400 246 to 7 793 for detector A, and 98 619 to 6 747 for detector B (4 324 665 and 7 439 respectively at zero actuation voltage), well above the DCR, resulting in 27.52 dB (detector A) and 11.65 dB (detector B) difference between the two extreme points. The detectors were biased as in the jitter measurement described above. Figure S7 shows the SNSPD detector counts under MEMS splitter actuation without normalization (the normalized values are in Fig. 1d). We attribute the large (44.6 times) difference in detector efficiency to the constriction in Detector B (see Fig. S2). We use this difference in detection efficiency as a property to extend the detection range of the power sensor.

V. MEMS DESIGN AND SIMULATIONS

We performed eigenmode simulations of the directional coupler supermodes using COMSOL Multiphysics at 795 nm wavelength of two Si_3N_4 ($n_{Si_3N_4} = 2$) coupled waveguides (width 400 nm, height 250 nm, horizontal separation 200 nm) clad by air ($n_{air} = 1$).

From coupled-mode theory, the power T at one of the output ports of a directional coupler. $n_{eff,i}$ is the effective index of the two supermodes (labeled by $i = 1, 2$, which depend on the MEMS vertical displacement y), and L_{wg} is the length of the coupling region

$$T_1 = \sin^2\left(\frac{(n_{eff,1}(y) - n_{eff,2}(y))\pi L_{wg}}{\lambda}\right), \tag{1}$$

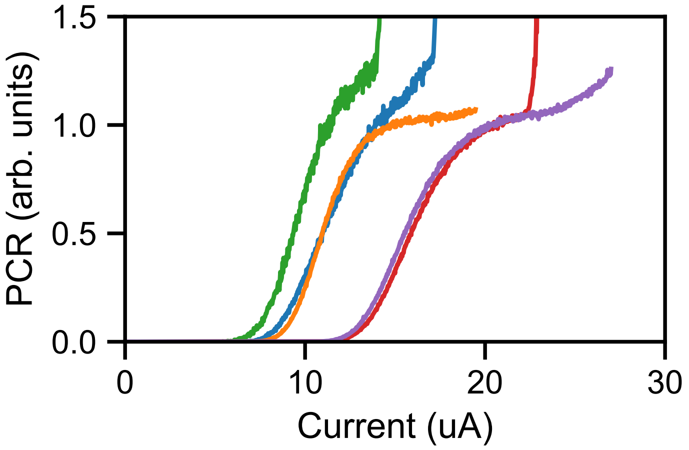

FIG. S4. Photon count rate normalized to the same range used during flood illumination at 850 nm. We characterized nominally identical devices after the release step (Figure S3e) at 2.5 K, and observed saturated internal quantum efficiency for 3 out of 5 devices (orange, purple, and red in the plot).

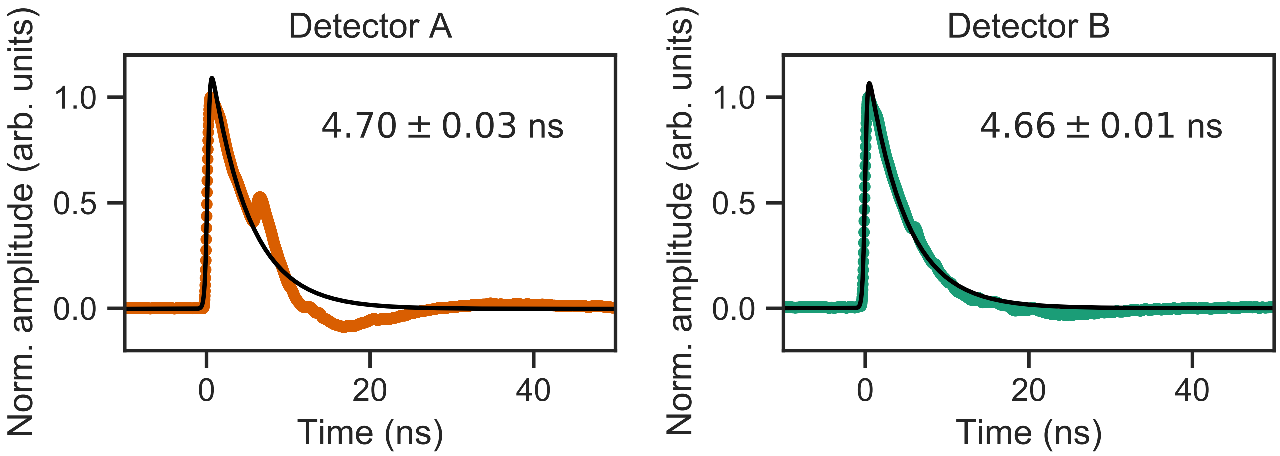

FIG. S5. Averaged detection pulse of our SNSPDs with reset times given by Gaussian decay fits of 4.73 ± 0.03 ns (detector A) and 4.73 ± 0.01 ns (detector B).

with $T_2 = 1 - T_1$ the power in the other port.

For the mechanical simulations, we assumed no initial stress with thickness 245 nm (combined Si_3N_4 and NbTiN films, assuming a minor etch of the Si_3N_4 due to finite selectivity of the HF underetch), and a Young's modulus of 180 GPa (reported Si_3N_4 values range from 120 GPa to 250 GPa). Initial capacitor separation was 3.535 µm (air bottom cladding plus Si_3N_4 thickness), and vacuum permittivity between the electrodes ($\epsilon = \epsilon_0$). The assumption of no initial stress holds for our system, since, although Si_3N_4 (and in our case, NbTiN) often presents significant internal tensile stress, single–clamped suspended structures like the actuators fabricated in our work relax film stress through strain (i.e. by contracting or expanding along the cantilever length), which drastically reduces their internal stress.

Using Hooke's law, the restoring force for an ideal spring is $F_k = ky$, with y vertical displacement. To calculate the spring constant k, our mechanical simulations follow the Euler-Bernoulli beam theory for a clamped-free beam (rectangular cross-section of width w, thickness t, length L, and for a material with Young's modulus E), with $k = \frac{3EI}{L^3}$, and its moment of inertia with respect to its neutral axis is $I = \frac{wt^3}{12}$, leading to

$$F_k = \frac{Ewt^3}{L^3}y. \tag{2}$$

This can be used to calculate the first resonance frequency as $f_r = \frac{1}{2\pi}\sqrt{\frac{k}{m}}$, with m the cantilever mass. Our

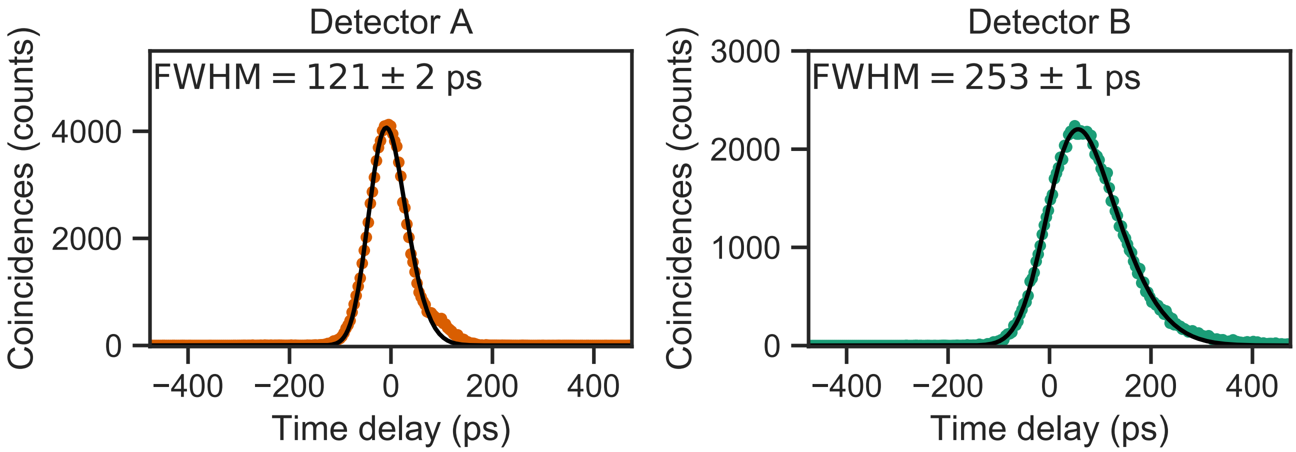

FIG. S6. Jitter curves for the two presented SNSPD devices with an exponentially modified Gaussian fit with a full width at half maximum of 121 ± 2 ps (detector A) and 253 ± 1 ps (detector B).

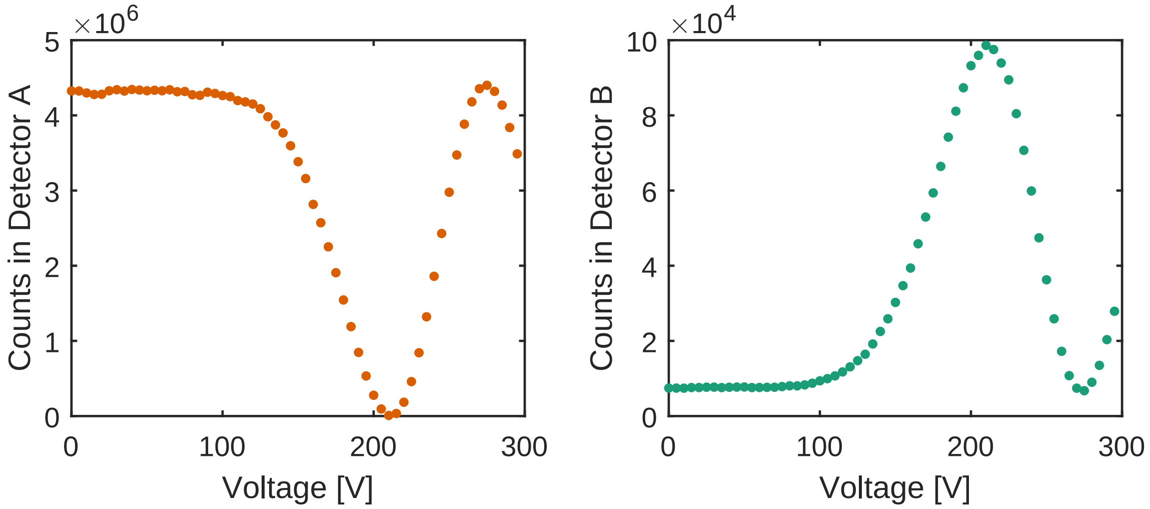

FIG. S7. Counts in Detector A and B under MEMS actuation.

calculations yield $f_r = 1.6\,\mathrm{MHz}$, in line with our measured value.

For the electrostatic force, we assume a parallel-plate capacitor of area wL. Assuming negligible fringing fields, the electrostatic force is

$$F_e = \frac{\varepsilon w L V^2}{2(y_0 - y)^2}. \tag{3}$$

The force balance combining Eqs. 2 and 3 yields a relation between actuation voltage and displacement.

$$V^2 = \frac{2Et^3 y (y_0 - y)^2}{4\varepsilon L^4}. \tag{4}$$

Figure S8a shows our simulated vertical displacement under MEMS voltage actuation.

Equation 4 can be solved analytically using Cardano's cubic solution, although for simplicity we solved it here numerically. Inserting the solution into Eq. 1 we obtain a relation between the power transmission and the actuation voltage $T_1(V)$, which we plot in Fig. S8b using our design parameters. Note that the initial coupling ratio in our simulation is set by our fabricated geometrical parameters, and the simulation assumes a constant directional coupler waveguide cross–section along the propagation direction for each actuator voltage.

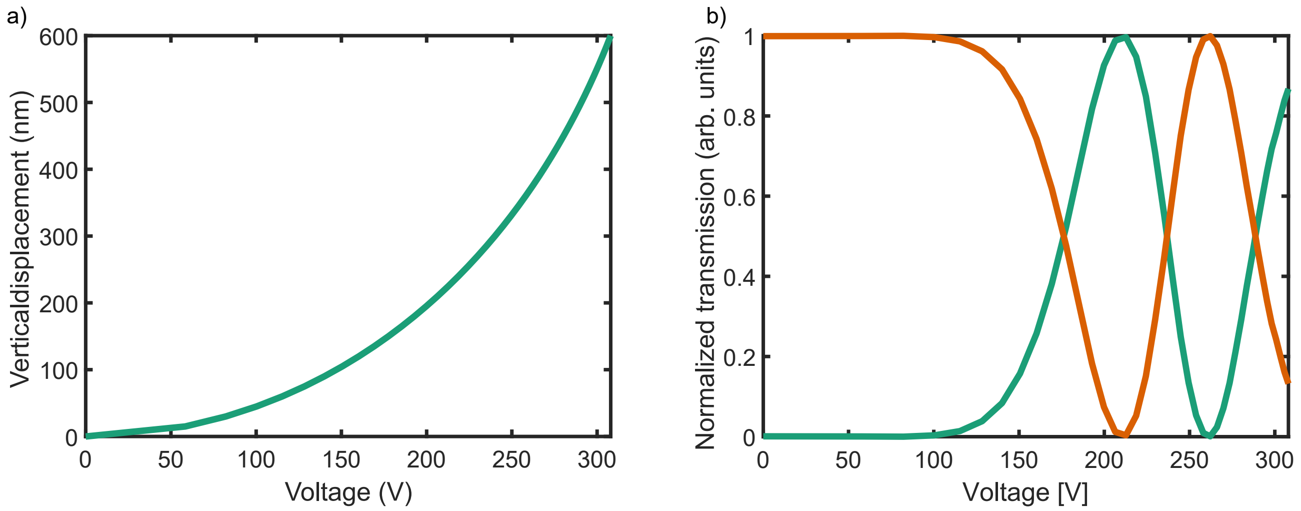

FIG. S8. Simulation results for our device. a) MEMS vertical displacement versus actuation voltage. b) MEMS beam splitter splitting ratio versus actuation voltage.

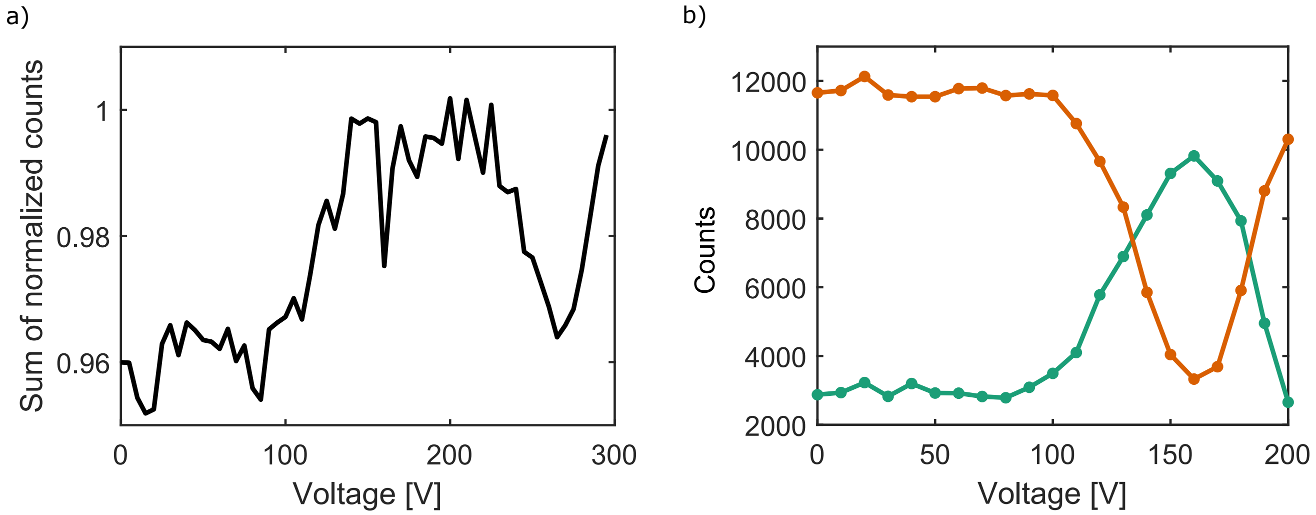

FIG. S9. MEMS splitter analysis and reference device measurement. a) Sum of normalized counts show an actuation-independent power conservation within 5%. b) Room temperature measurements of a grating–coupled MEMS splitter on the same chip show symmetric operation with similar counts, further demonstrating that the difference in measured optical power can be attributed to the SNSPDs and not to the MEMS splitter.

VI. MEMS SPLITTER ANALYSIS

To investigate the cause of the difference in efficiency in our two detectors, we investigate the power balance of our design. Figure S9a shows the sum of the normalized tuning curves in our device. The sum being within 5% of unity indicates that power is conserved during actuation, and thus MEMS tuning does not induce the large difference between SNSPD efficiencies of 44.6 times. To further prove this, and to demonstrate that the efficiency mismatch is not intrinsic to our MEMS splitter, we performed room temperature characterization of a similar MEMS splitter on the same chip that features grating–coupled input and output ports. The results, shown in Fig. S9b demonstrate symmetric tuning without count normalization. The lower extinction observed in this device is attributed to the difference in operation temperature, which results in a change in device geometry via relative thermal expansion of the materials forming the cantilever.

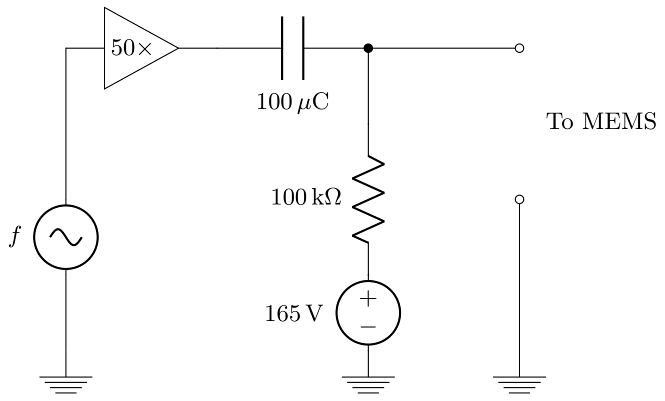

FIG. S10. Circuit for the frequency measurement. The measured SSPD counts where fitted to a sine and the amplitude used as frequency response in Fig. 2a.

VII. MEMS FREQUENCY RESPONSE MEASUREMENT

We actuated the MEMS system using a function generator with an amplifier (amplification factor 50) as explained above. We operated the MEMS in a bias point of 165 V with an amplitude of 60 V using an in-house built high–voltage bias tee (See Fig. S10). The trigger linked to the sinusoidal excitation and the detection event of the on–chip SNSPD was connected to a time correlator (qutag, qutools). The time–tagged data was evaluated using our in-house built software, ETA [4].

The measurements at the frequencies of 1 MHz and 2 MHz were largely amplified by the mechanical resonance, which led to a large waveform distortion (i.e. effectively actuation with amplitudes higher than our measured actuation curve in Fig. 1d) and the SNSPD response fluctuates between maximum and minimum transmission. Due to the lack of a characterization curve covering such large amplitudes, we were unable to fit an analytical model to their time response, and their amplitude in Fig. 2a is the difference between maximum and minimum transmission, and thus represents a lower bound to their amplitude. This waveform distortion, caused by a large mechanical amplification near the resonance, hampers our measurement of the exact resonance frequency and mechanical Q-factor. We expect the Q-factor to be high due to the absence of viscous damping and the low coefficients of thermal expansion at cryogenic temperatures resulting in low thermo–elastic loss.

At frequencies above 10 kHz we observe detection events on an unbiased SNSPD channel due to the large amplification on the readout chain. This is much more significant on detector B due to the low critical current, that also requires a lower trigger level. Figure S11 shows these counts for both detectors with a sinusoidal actuation between 10 kHz and 5 MHz, an amplitude of 60 V at a working point of 165 V. Through careful RF engineering on the electrodes on chip (e.g. ground planes encapsulating the signal lines) this cross-talk can be reduced or eliminated. We investigated the behavior for DC actuation and we observe no additional dark counts generated by the biasing of the MEMS actuator (Figure S12)

VIII. MEMS SPLITTER STABILITY AND HYSTERESIS

We measured the device stability by setting the MEMS actuator to 190 V during 60 min, and the measurement can be found in Fig. S13a. Our measurement yields constant transmission within a standard deviation below 0.5 %. The small asymmetric instability is most likely caused by setup drift (e.g. laser power or polarization fluctuations, fiber and setup vibrations) and not MEMS instability, since the cantilevers feature a high stiffness, and fluctuations would most likely happen near the MHz–range fundamental resonance.

We then performed a hysteresis measurement (Fig. S13b) by cycling the MEMS actuator voltage between 120 V and 240 V over 10 cycles at a frequency of 1 Hz, yielding a hysteresis below 2.4 % in optical transmission. This is likely an overestimated value, since this measurement will be affected by power fluctuations caused by free–space coupling and mechanical vibrations in the measurement setup.

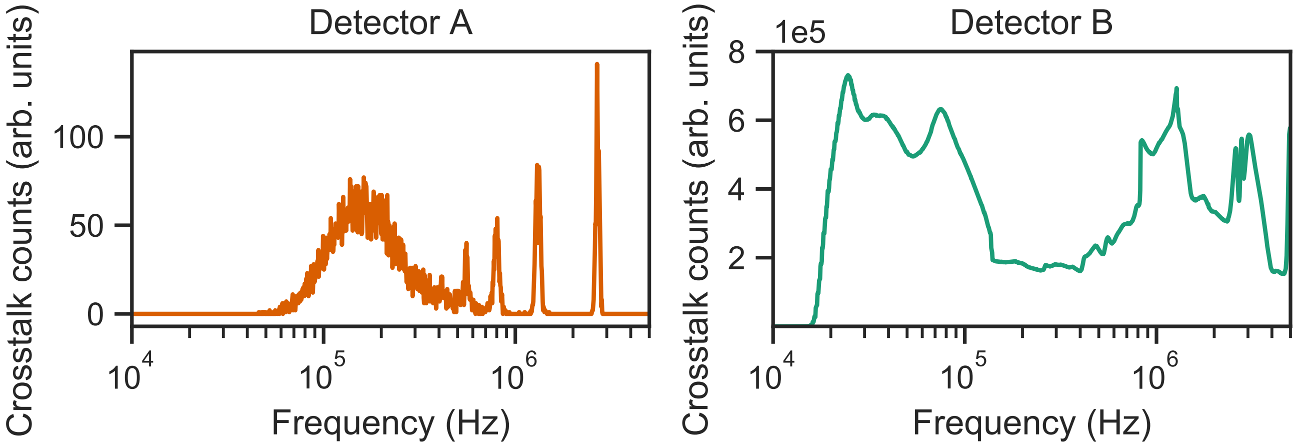

FIG. S11. Detection counts generated by sinusoidal actuation (from 10 kHz to 5 MHz with an amplitude 60 V at a working point 165 V) of the MEMS on the unbiased SNSPD readout channel.

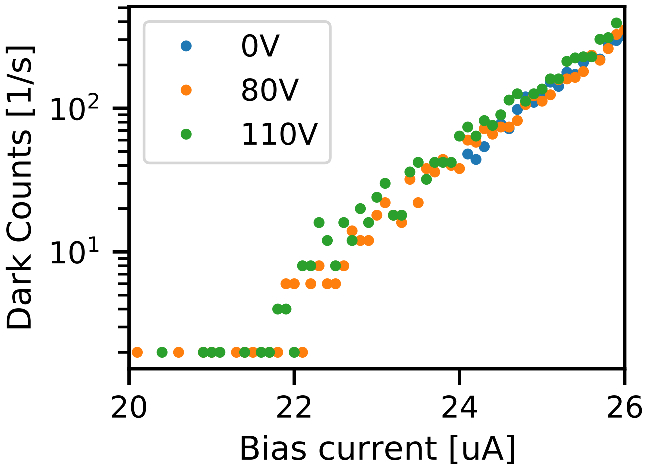

FIG. S12. Dark counts under 0 V, 80 V and 110 V static actuation. This data was measured in a pre-characterization experiment and no change in dark counts was observed.

IX. POWER CONSUMPTION

Electrical energy (E_c) from charging and discharging a capacitor with capacitance C is $E_c = CV^2$, which for a driving frequency of f translates into a power of $P = fCV^2$. Compared to charge–discharge power, the contribution of leakage currents in such large capacitors is negligible (see discussion below). The capacitance of our device can be estimated from a parallel–plate capacitor with its area dominated by the 6400 µm^2 contact pad and a SiO_2 ($\varepsilon = 3.9$) gap of 3 µm. For an AC voltage actuation of 100 V on top of a DC bias of 100 V to cover the full splitting ratio range, and an actuation frequency of 100 kHz (right below the resonance), the power consumption amounts to less than 75 µW. Our circuit comprises the capacitance of our actuator followed by series resistors from the electrical wiring and connections, and the voltage source. Since the on–chip wirebonds are made by superconducting aluminum and the PCB features a resistance below 0.1 Ω, the heat dissipation likely occurs in the high-resistivity voltage source (10 MΩ). We note that for lower–voltage MEMS actuator design, such as those reviewed in Ref. [5], the AC power consumption is usually in the nW range due to a capacitance not dominated by the contact.

The power consumption due to leakage current can be estimated using the resistance of the dielectric between the parallel plates as $P_{leak} = V^2/R$. The high resistivity of SiO_2 and of vacuum leads to a negligible leakage currents, and thus to power dissipation below 10 fW. In a large-scale PIC based on these actuators, however, the power consumption

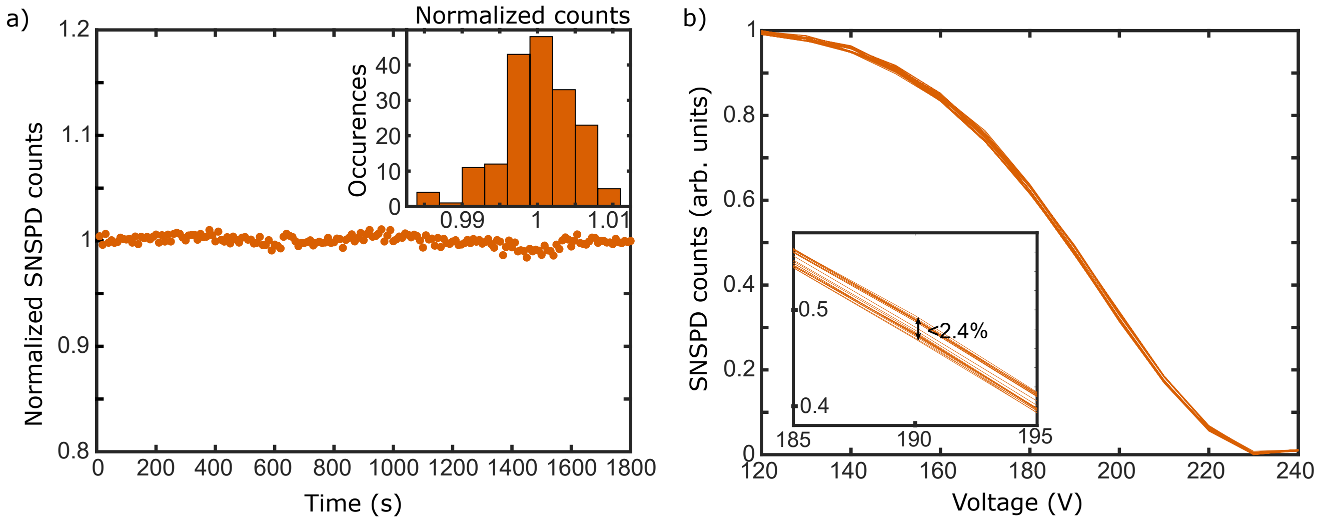

FIG. S13. Stability and hysteresis of our device. a) Stability measurement with our MEMS beam splitter under 190 V actuation during 60 min. Inset: distribution of the measured SNSPD counts, yielding a standard deviation below 0.5 %. b) Hysteresis measurement consisting of periodic charge–discharge cycles of the MEMS actuator between 120 V and 240 V over 10 cycles, showing hysteresis below 2.4 %.

will be most likely dominated by the control electronics and the routing of electrical lines.

X. HIGH–DYNAMIC RANGE DETECTOR

We illuminated our device using a continuous wave laser at 795 nm and swept the input power using a fiber–coupled attenuator while recording the SNSPD counts per second. To reach the wide range of input power we additionally added absorption based attenuators (−130 dB, −120 dB, −70 dB, −40 dB, −10 dB) into the free–space path. The different input power regions per leg are aligned by matching the slope of the measured detection counts based on a linear fit. We connected the measurements at the switching points of our high–dynamic range detector by keeping the input power constant while ramping the MEMS actuation voltage between 0 V and 196.5 V. The power axis is normalized to the maximum measurable power (3.15 µW at the cryostat window) by the power sensor. This leads to an efficiency of −33.7 dB for detector A and −49.26 dB for detector B, when all the power is routed to the respective detectors.

XI. POWER STABILIZATION MEASUREMENTS

A in–house built digital–to–analog converter (DAC) circuit and the SNSPD driver were controlled by our in–house built LabView based laboratory control system. The output of the DAC was amplified using the high–voltage high–speed amplifier as described in the previous section and fed as offset voltage to the high–voltage power supply (Keithley 2410) (See Fig. S14). The current detection count rate measured by the SNSPD driver was read out every 100 ms (currently limited by the counting electronics) and a PID control loop stabilized the applied voltage with the photon detection events as the process variable. We tested the control loop by ramping the input power from 0 µW to 300 µW with 1 $\mu W\,s^{-1}$.

XII. SINGLE–PHOTON MEASUREMENTS

The quantum dot sample was grown at Johannes Kepler University Linz via molecular beam epitaxy by etching nanoholes into an AlGaAs layer before filling the holes with GaAs [6]. The quantum dot is embedded in a λ–cavity with a ditributed Bragg reflector consisting of 9 thin film pairs (GaAs/AlGaAs) below and 2 pairs above the cavity. A solid immersion lens was placed on top of the sample to increase light extraction efficiency further. The sample was placed inside a closed–cycle Helium–cryostat operating at 5 K and emitted light was collected and collimated by

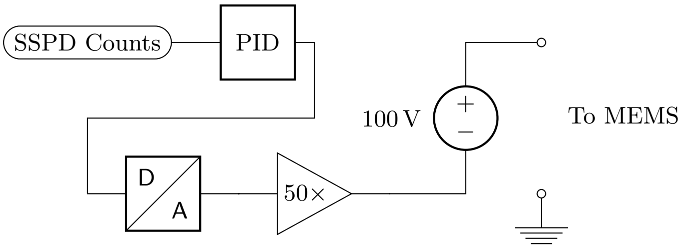

FIG. S14. Circuit for feedback loop stabilizing the count rate measured by the SNSPD Driver on the SSPD. The PID controller was implemented in LabView.

an aspheric lens inside the cryostat with a numerical aperture of about 0.5. The light was coupled into the collection path via a 90:10 beam splitter (BS) in order to minimize losses and the excitation laser was filtered out with three tunable notch filters (NF) with a bandwidth of 0.4 nm. An optical fiber was used both as a pinhole to further suppress the excitation laser and to route the light to a transmission spectrometer (TG). Here, biexciton and exciton emission were coupled into separate optical fibers.

The exciton emission was split at 50:50 fiber beam splitter and both arms were connected to two superconducting nanowire single–photon detectors (Single Quantum EOS) with efficiencies of 50 % and 60 %, timing jitters of 20 ps and 30 ps, and dark count rates of 0.006 cts/s and 0.017 cts/s.

In the measurement with an on–chip detector, one of the arms was coupled to the detectors on–chip to measure the life time of the QD fully on–chip. The HOM measurement is measured with a commercial detector off–chip and one of the detectors on–chip.

We extract the lifetime by fitting the fit function (Eq. 5) including the Instrument Response Function (IRF) (see Fig. S6) of the detector.

$$f(t) = A \cdot \mathrm{IRF}(t) * \left(\mathrm{H}(t) \cdot \exp\left(\frac{-t}{\tau_{XX}}\right) * \mathrm{H}(t - t0) \cdot \exp\left(\frac{-(t - t0)}{\tau_X}\right)\right) \tag{5}$$

τ_X is the exciton lifetime, H is the Heaviside function and $*$ denotes the linear convolution with multiplication taking precedence. Due to the X being fed by the XX decay, an additional decay with a fixed lifetime $\tau_X X$ extracted from supporting measurements is part of the fitting function. The fit is made using LMFIT [7] and the uncertainty given for the parameters is estimated from the fit quality.

[1] Yunpeng Zhu, Jie Wang, Weiqiang Xie, Bin Tian, Yanlu Li, Edouard Brainis, Yuqing Jiao, and Dries Van Thourhout, "Ultra-compact silicon nitride grating coupler for microscopy systems," Optics Express **25**, 33297–33304 (2017).

[2] Lu Zhang, Lixing You, Liliang Ying, Wei Peng, and Zhen Wang, "Characterization of surface oxidation layers on ultrathin NbTiN films," Physica C: Superconductivity and its Applications **545**, 1–4 (2018).

[3] Archan Banerjee, Robert M. Heath, Dmitry Morozov, Dilini Hemakumara, Umberto Nasti, Iain Thayne, and Robert H. Hadfield, "Optical properties of refractory metal based thin films," Optical Materials Express **8**, 2072–2088 (2018).

[4] Zuzeng Lin, Lucas Schweickert, and Samuel Gyger, "Timetag/ETA: ETA 0.7.7," Zenodo (2020).

[5] Carlos Errando-Herranz, Alain Yuji Takabayashi, Pierre Edinger, Hamed Sattari, Kristinn B. Gylfason, and Niels Quack, "MEMS for Photonic Integrated Circuits," IEEE Journal of Selected Topics in Quantum Electronics **26**, 1–16 (2020).

[6] Massimo Gurioli, Zhiming Wang, Armando Rastelli, Takashi Kuroda, and Stefano Sanguinetti, "Droplet epitaxy of semiconductor nanostructures for quantum photonic devices," Nature Materials **18**, 799–810 (2019).

[7] Matthew Newville, Till Stensitzki, Daniel B. Allen, and Antonino Ingargiola, "LMFIT: Non-Linear Least-Square Minimization and Curve-Fitting for Python," Zenodo (2014).
